# Advanced Nanobiocomposite Hydrogels Incorporating Organofunctionalized LDH for Soft Tissue Engineering Applications

**DOI:** 10.3390/polym17040536

**Published:** 2025-02-19

**Authors:** Ionut-Cristian Radu, Eugenia Tanasa, Sorina Dinescu, George Vlasceanu, Catalin Zaharia

**Affiliations:** 1 Advanced Polymer Materials Group, Faculty of Chemical Engineering and Biotechnology, National University of Science and Technology POLITEHNICA Bucharest, 1-7 Gh. Polizu Street, 011061 Bucharest, Romania; ionut_cristian.radu@upb.ro (I.-C.R.); vlasceanu.georgemihail@yahoo.ro (G.V.); 2 Department of Physics, Faculty of Applied Sciences, National University of Science and Technology POLITEHNICA Bucharest, 011061 Bucharest, Romania; eugenia.vasile@upb.ro; 3 National Research Center for Micro and Nanomaterials, National University of Science and Technology POLITEHNICA Bucharest, 313 Splaiul Independenţei, 060042 Bucharest, Romania; 4 Department of Biochemistry and Molecular Biology, University of Bucharest, 050095 Bucharest, Romania; sorina.dinescu@bio.unibuc.ro; 5 Research Institute of the University of Bucharest, 050663 Bucharest, Romania

**Keywords:** hydrogel, nanocomposite, clay, LDH, (HEMA/AMPSA) copolymers

## Abstract

Nanocomposite hydrogels are gaining significant attention for biomedical applications in soft tissue engineering due to the increasing demand for highly flexible and durable soft polymer materials. This research paper focused on investigating and optimizing a procedure for the development of novel nanocomposite hydrogels based on poly(2-hydroxyethyl methacrylate)-co-(2-acrylamido-2-methylpropane sulfonic acid) (HEMA/AMPSA) copolymers. These hydrogels were synthesized through a *grafting-through* process, where the polymer network was formed using a modified clay crosslinker. The layered double hydroxide (LDH) clay modified with 3-(trimethoxysilyl)propyl methacrylate (ATPM) was synthesized using a novel recipe through a two-step procedure. The nanocomposite hydrogel compositions were optimized to achieve soft hydrogels with high flexibility. The developed materials were analyzed for their mechanical and morphological properties using tensile and compressive tests, transmission electron microscopy (TEM), scanning electron microscopy (SEM), and micro-computed tomography (micro-CT). The swelling behavior, network density, and kinetic diffusion mechanism demonstrated the specific characteristics of the materials. The modified LDH-ATPM was further characterized using Thermogravimetry (TGA), FTIR-ATR and X-ray diffraction (XRD). Biological assessments on human adipose-derived stem cells (hASCs) were essential to evaluate the biocompatibility of the nanocomposite hydrogels and their potential for soft tissue applications.

## 1. Introduction

The chemical integration of an organic phase, in the form of a polymer matrix, with an inorganic phase, such as nanoparticles or layers, has been shown to enhance the properties of traditional polymer networks. These characteristics are particularly evident in inorganic agents, leading to the development of novel polymer materials with enhanced wproperties and added value [1,2,3,4,5,6,7]. In essence, the proposed association addresses the inherent mechanical weaknesses, brittleness, and dimensional instability exhibited by polymer gels in their swollen state. Polymer hydrogel typically consists of a 3D network with a high capacity for water absorption, making it a soft and wet material in its swollen state. As a result, it exhibits properties of both solids and liquids. However, current chemical crosslinking methods using traditional low-molecular-weight agents have yet to produce hydrogels with the advanced properties required for certain specialized medical applications. Consequently, the field of polymer nanomaterials has shifted its focus toward addressing these limitations. A variety of hybrid materials incorporating inorganic agents such as silica, titania, clay, or graphene oxide have been extensively investigated and developed [6,8,9,10,11,12,13]. The method employed for synthesizing these materials typically involved an in-situ sol-gel reaction with inorganic nanoparticles that had been pre-modified with organic agents [14,15]. In recent years, the field of nanocomposites based on polymer–clay gels has undergone rapid development due to their unique structural and mechanical properties. These layered nanomaterials possess the ability to achieve exceptional structural flexibility, enabling the creation of innovative hybrid materials with controlled functionalities and enhanced mechanical properties. The incorporation of clay nanoparticles into polymeric matrices has been shown to significantly improve tensile strength, elasticity, and toughness by promoting strong interfacial interactions and uniform dispersion within the hydrogel network [8,16,17,18,19,20,21]. Numerous studies have highlighted that the mechanical reinforcement of nanobiocomposite hydrogels is influenced by various factors, including the type of clay, surface modifications, compatibility with the polymer matrix, and processing parameters. Notably, hydrogels incorporating montmorillonite (MMT) or halloysite nanotubes (HNTs) exhibit superior mechanical performance, as these nanofillers create a percolating network within the polymer matrix, facilitating stress transfer and enhancing structural resilience [22,23,24]. Furthermore, the application of multifunctional crosslinking strategies, such as peroxide-initiated clay surface modifications, further reinforces the hydrogel network, resulting in enhanced compressive and tensile properties [25,26]. A unique network microstructure was developed, incorporating a small amount of inorganic clay that also functions as cross-linkers. These clay types and other agents can be functionalized to increase the interaction with the organic phase by extended homogenization [25,26,27,28,29]. Achieving a homogeneous dispersion of inorganic clay layers within the polymer matrix is the key approach required for nanocomposites to demonstrate superior mechanical properties. Achieving this objective requires the design of exfoliated nanocomposite structures, where the clay layers are separated or delaminated, resulting in essentially individual dispersions. In developing such structures, it is crucial to consider the compatibility of the clay, which can be enhanced through modification with organic agents, as well as the inherent interactions between the polymer matrix and the clay. Layered double hydroxides (LDHs) represent a class of two-dimensional nanostructured materials characterized by positively charged brucite-like layers. These layers are typically formed through the partial substitution of divalent metal cations (Mg^2+^, Zn^2+^) with trivalent metal cations (Al^3+^, Fe^3+^), resulting in a nanoscale-thin structure. To maintain charge neutrality and structural stability, interlayer spaces are occupied by various anions, such as carbonate, nitrate, or organic species, which contribute to the electrostatic equilibrium of the material [27,30,31]. These nano-layered inorganic materials have demonstrated excellent properties over time, including high interlayer anion exchange capacity, cost-effectiveness, strong interfacial interactions, good biocompatibility, a high specific surface area, and the ability to undergo various surface modification reactions [32,33,34,35,36,37,38]. The remarkable properties of LDHs have made them highly valuable in various biomedical applications, including controlled drug release formulations and tissue engineering [39,40,41]. The development of nanocomposite hydrogels combines the unique features of each constituent component, enabling the creation of advanced materials with the necessary capabilities for applications in tissue engineering.

Tissue engineering involves the careful selection of materials, cells and inductors that are appropriate for the type of application required. Such materials play an important role in soft tissue engineering applications to meet the needs of the affected tissue. Selected materials must support tissue regeneration by promoting cellular adhesion, proliferation and differentiation. It is also important to demonstrate good biocompatibility and to mimic the in vivo properties of the tissue as closely as possible [42]. Depending on the application, different cell types could be used. Adipose tissue-derived stem cells (ASCs) are of great interest due to their potential to differentiate into multiple types of lineages (osteogenic, adipogenic, chondrogenic, etc.) and are easily obtained from lipoaspirates [13,43,44,45]. The development of new biocompatible scaffolds for tissue engineering could greatly improve the treatment options for patients in need of post-mastectomy reconstruction, skin regeneration or reconstruction of other soft tissue defects [42,46]. The potential use of various types of hydrogels based on poly(2-hydroxyethyl methacrylate) was investigated and confirmed for biomedical applications, such as breast reconstruction, soft tissue augmentation [47], skin tissue engineering [48], bone [49] and cartilage tissue engineering [50]. Furthermore, poy(2-acrylamido-2-methylpropane sulfonic acid) hydrogels developed by Hu et al. [51] promoted cellular adhesion and indicated a potential use for soft tissue engineering. While LDH has been previously studied for its applications in bone tissue engineering [52,53,54], our research underscores its potential for use in soft tissue engineering as well.

This paper builds upon previous research studies in the field, aiming to develop innovative approaches in the domain of nanocomposites. The development of directly cross-linked network nanocomposites based on modified LDH-ATPM is essential to address the challenges in this field, a goal that cannot be achieved using traditional cross-linking agents. This research focuses on creating individually exfoliated nanocomposite structures of LDH layers within a Poly(HEMA-co-AMPSA) matrix, offering superior mechanical flexibility, reduced brittleness, and enhanced dimensional stability—key properties required for applications in soft tissue engineering. Furthermore, the developed nanocomposite hydrogels are designed to exhibit a porous structure that mimics the extracellular matrix, enabling superior interactions with cellular components. To achieve this, a novel LDH clay structure was developed by considering different ion types and selecting an appropriate polymer matrix.

## 2. Materials and Methods

The monomers 2-hydroxyethyl methacrylate (HEMA) and 2-acrylamido-2-methylpropanesulfonic acid (AMPSA), along with the crosslinking agent N,N′-methylene-bis-acrylamide (MBA) and the initiator potassium persulfate (KP), were sourced from Sigma Aldrich. The reagents used for the synthesis of the modified clay included Al(NO_3_)_3_·9H_2_O, Co(NO_3_)_2_·6H_2_O, sodium dodecyl sulfate (SDS), sodium hydroxide, 3-(trimethoxysilyl)propyl methacrylate (ATPM), and hexadecyltrimethylammonium bromide (CTAB). All reagents were supplied by Sigma Aldrich (3050 SPRUCE Street, St. Louis, MO 63103, USA).

### 2.1. Synthesis of Modified Layered Double Hydroxide (LDH-ATPM)

The synthesis of modified layered double hydroxide (LDH-ATPM) involved a two-step reaction process. The first step consisted of the organophilization of LDH-SDS, performed using a previously established method [55]. In this step, the reactants were substituted with CoCl_2_·6H_2_O and AlCl_3_·6H_2_O. The second step involved the modification of LDH-SDS using ATPM, a silane-acrylate modifier agent, following a previously established method [55].

### 2.2. Preparation of HEMA-AMPSA/LDH-ATPM Nanocomposite Hydrogels

The nanocomposite materials were synthesized through in situ sol-gel free radical polymerization of HEMA and AMPSA monomers. The polymerization process was initiated by potassium persulfate at 60 °C in the presence of dispersed LDH-ATPM, without the addition of a cross-linking agent. This method was employed as a comparison to previously reported research [55,56]. Monomer mixture solutions were prepared with HEMA-AMPSA molar ratios of 100:0, 97:3, and 95:5. It was observed that a higher AMPSA ratio resulted in reduced mechanical stability. The monomer solutions were then combined with dried, modified LDH-ATPM in concentrations of 1% and 2% (*w*/*v*) relative to the solution volume. However, a higher amount of modified LDH-ATPM clay exhibited poor dispersion in the monomer solution, providing no significant advantages to the network structure or mechanical properties. The total monomer molar concentrations in the synthesis solutions were 5 mol/L and 2 mol/L. For the 5 mol/L concentration, all ratios (100:0, 97:3, and 95:5 HEMA-AMPSA mol/mol) were prepared, whereas for the 2 mol/L concentration, only the 97:3 and 95:5 HEMA-AMPSA (mol/mol) ratios were obtained. Notably, the 100:0 HEMA-AMPSA (mol/mol) ratio at the 2 mol/L concentration did not exhibit any cross-linking reaction. The prepared samples are summarized in Table 1. Additionally, similar samples were prepared using MBA as a cross-linking agent, with the same monomer ratios and molar concentrations (100:0, 97:3, 95:5 for 5 mol/L and 97:3, 95:5 for 2 mol/L) but without the inclusion of modified LDH-ATPM. These samples were created to facilitate a comparison of network density. The MBA molar ratio relative to the monomers ranged from 1% to 3%.

### 2.3. Preparation of Lyophilized Nanocomposite Samples

The nanocomposite hydrogels underwent a lyophilization process to prepare samples suitable for biological assays. This process involved freezing the hydrogel samples at −20 °C for 24 h, followed by water sublimation for 48 h.

### 2.4. Fourier Transform Infrared-Attenuated Total Reflectance (FTIR-ATR) Analysis

The physico-chemical characterization of the nanocomposite hydrogels and modified clay was conducted using FTIR analysis with a Bruker Vertex 70 FT-IR spectrophotometer equipped with an Attenuated Total Reflectance (ATR) accessory. The FTIR spectrophotometer performed 32 scans at a resolution of 4 cm^−1^ within the mid-IR region of 4000–600 cm^−1^.

### 2.5. X-Ray Diffraction (XRD) Analysis

The X-Ray diffraction patterns of clay species including crude LDH, LDH-SDS and LDH-ATPM were carried out with a Panalytical X’PERT MPD X-ray Diffractometer, in the range 2θ = 10–80. An X-ray beam characteristic to Cu Kα radiation was used (α = 1.5418 Å).

### 2.6. Evaluation of Clay Modification via Titration

This approach was conducted using a titration procedure specific for quantifiable hydroxyl bonds, in accordance with international standard D4274-99. The procedure involved preparing a mixed titration solution of phthalic anhydride and pyridine, followed by the addition of clay. The final solution was heated at 105 °C for 1 h. After the reaction, the mixture was titrated with freshly prepared 0.1N NaOH solution (with a predetermined factor based on its reaction with oxalic acid) in the presence of phenolphthalein as an indicator. A blank solution containing the same amount of phthalic anhydride and pyridine (without clay) was used as a control. The procedure was performed in duplicate for accuracy.

### 2.7. Swelling Behavior and Kinetics

The purpose of the analysis was to evaluate the network density of the nanocomposite hydrogels and the kinetic behavior of their volume change upon exposure to moisture. The procedure involved immersing dried samples, previously maintained at a constant mass, in a phosphate buffer solution (PBS) with a pH of 7.4 at room temperature. The mass of each sample was measured at five-minute intervals during the first hour and at 15-min intervals for the following hours. The experiment was conducted in triplicate to ensure accuracy and reliability.

### 2.8. Thermogravimetric Analysis (TGA)

To assess the thermal stability of the naocomposite hydrogels, thermogravimetric analysis (TGA) was conducted using a Netzsch TG 209 F1 Libra instrument. The procedure involved heating the sample from 25 °C to 700 °C at a rate of 10 K/min under a nitrogen atmosphere.

### 2.9. Morphological Characterization

*Scanning electron microscopy (SEM*). The morphology and surface porous structure of the nanocomposite hydrogels were evaluated using a Quanta Inspect F Scanning Electron Microscope (SEM) (FEI, The Netherlands), equipped with a 1.2 nm resolution field emission gun (FEG) and an X-ray energy dispersive spectrometer (EDS).

*Transmission electron microscopy (TEM).* The dispersion of clay layers and the intercalated/exfoliated structures were analyzed using transmission electron microscopy (TEM) with a TECNAI F30 G2 S-TWIN microscope operated at 300 kV, featuring an Energy Dispersive X-ray Analysis (EDAX) facility.

*Computer Tomography (micro-CT).* Micro-CT analysis was conducted using a Bruker SkyScan 1272 microCT scanner. Each sample was scanned at a voltage of 50 kV and a current of 200 μA, without the use of any filter. Images were acquired with a resolution (pixel size) of 2.75 µm, a rotation step of 0.2°, and an average of 5 frames per capture. Image reconstruction was performed using NRecon 1.7.1.6 software from Bruker microCT, with the following parameters: postalignment: 4, smoothing: 2, beam hardening correction: 55%, and ring artifact correction: 11. CTvox 3.3.0 r1403 software from Bruker microCT was utilized for image processing.

### 2.10. Mechanical Investigation of the Nanocomposite Hydrogels

Mechanical analyses were conducted using an Instron 2519-107 Universal Testing Machine equipped with a 5 kN load cell. Tensile tests were performed at ambient temperature on dumbbell-shaped specimens, with a tensile rate of 10 mm/min. Compression tests were also conducted on nanocomposite hydrogel samples (10 mm in diameter and 5 mm in height) using the same testing machine. The compression tests were carried out at room temperature with a compression rate of 1 mm/min. Both tensile and compression tests were performed exclusively on hydrogels with a 2 mol/L concentration.

### 2.11. Rheological Measurements

Rheological measurements were performed using a Kinexus Pro Malvern rotational rheometer equipped with a temperature control unit and a 20 mm diameter parallel plate geometry. The samples were analyzed over a frequency range of 1 to 30 Hz. All measurements were conducted exclusively on hydrogels with a 2 mol/L concentration.

### 2.12. Biocompatibility Assessment of the Materials

Human stem cells derived from adipose tissue (hASCs, ThermoFischer Scientific, Whaltam, MA, USA, R7788115) were cultured and maintained in DMEM media (Sigma-Aldrich, Darmstadt, Germany) supplemented with 10% fetal bovine serum (FSB, ThermoFischer Scientific, Whaltam, MA, USA) and 1% antibiotic antimycotic solution (Sigma-Aldrich, Darmstadt, Germany). The cells were then seeded onto the materials (2 mol/L), and their biocompatibility was assessed by quantitative and qualitative assays after 2 and 5 days of cell culture. The quantitative MTT test (Sigma Aldrich, Darmstadt, Germany) was used to evaluate cell viability and proliferation rate in contact with the materials. The cells that had been seeded onto the materials were exposed to 3-(4,5-dimethylthiazol-2-yl)-2,5-diphenyltetrazolium bromide for a period of four hours, after which the live cells metabolised the compound to formazan crystals. The formazan crystals were then solubilised in isopropanol, after which the solution’s absorbancy was measured at 550 nm by spectrophotometry.

The level of toxicity induced by the materials when put in contact with cells was evaluated using a quantitative test LDH (Tox7, Sigma-Aldrich, Darmstadt, Germany). After 2 and 5 days of cell culture, the levels of lactate dehydrogenase released by dead cells in the media were measured. Following the kit instructions, the solution’s absorbances were measured at 490 nm.

A qualitative Live/Dead test (from ThermoScientific, Whaltam, MA, USA) was used to stain and visualise the live cells (calcein AM, green) and nuclei of dead cells (ethidium homodimer, red) after 2 and 5 days of cell culture by fluorescence microscopy (IX-73 Olympus).

## 3. Results and Discussion

### 3.1. Evaluation of Clay Modification by Titration

The evaluation of clay modification with silane double bonds was one of the primary objectives of this research. This assessment aimed to determine the nanocomposite network density and its correlation with mechanical properties and swelling behavior. However, it was observed that the relative cross-linking density, determined indirectly through the swelling degree method, was insufficient for accurately estimating the number of possible double bonds per unit mass of the modified clay. The results of this procedure revealed that unmodified clay (LDH-SDS) exhibited approximately 1 mol of hydroxyl groups per gram of clay, whereas modified clay (LDH-ATPM) exhibited approximately 0.7 mol of hydroxyl groups per gram of clay. The number of double bonds introduced by the silane agent can be estimated by considering: (i) the difference of 0.3 mols between the consumption of hydroxyl groups by the silane agent and the number of hydroxyl groups available; and (ii) the bonding possibility of the silane agent with clay between one and three bonds. This approach is supported by the reactivity of the clay, which contains three methoxy groups capable of undergoing reactions. On average, two bonds were considered. Consequently, it can be deduced that the consumption of hydroxyl groups by the silane agent is equivalent to 0.15 mol. In summary, each gram of modified clay contained 0.7 mol of hydroxyl groups and 0.15 mol of double bonds, introduced by the 0.15 mol of silane agent, which consumed 0.3 mol of hydroxyl groups. These results enabled the estimation of the molar quantity of double bonds present in each nanocomposite hydrogel sample. Specifically, the nanocomposite hydrogel samples contained 0.15 mol of double bonds per 0.2 mol of monomers for 1% LDH-ATPM and 0.3 mol of double bonds per 0.2 mol of monomers for 2% LDH-ATPM. In the case of MBA, the highest molar ratio considered was 3% due to increased rigidity and sample cracking. At this 3% molar ratio, MBA contributed 0.006 mol of cross-linking agent, introducing 0.012 mol of double bonds. Compared to the MBA agent, the LDH-ATPM samples exhibited a higher ratio of available double bonds for cross-linking.

### 3.2. Swelling Degree and 3D Nanocomposite Network Design

The swelling behavior of the synthesized nanocomposite hydrogel was evaluated to determine its network structure and cross-linking density. Building on previous research [55], where a mixture of LDH-ATPM and the conventional cross-linker MBA was used, the present study demonstrated the formation of unique 3D network structures using only LDH-ATPM. As previously mentioned, the 100:0 HEMA-AMPSA (mol/mol) ratio at a 2 mol/L concentration showed no cross-linking reaction. This was likely due to the low amount of monomers, which rendered the monomer phase unable to adequately disperse the high LDH-ATPM content throughout the solution. Such dispersion is essential for effective cross-linking and the formation of a proper 3D network. Due to its modification with a silane-acrylate agent, the LDH-ATPM acquired a more organic nature. As a result, the solvent water molecules alone were unable to effectively disperse the clay particles without the assistance of monomer molecules. All samples were subjected to swelling tests by immersion in phosphate buffer solution (PBS) at a pH of 7.45 and a temperature of 37 °C. The correlation between swelling degree and cross-linking density for conventional cross-linking agents is well-documented in the existing literature [57,58]. A direct correlation has been demonstrated between the quantity of the cross-linking agent, expressed as molar concentration, and the resulting swelling behavior. For MBA, the known molar concentration enables the determination of the molar concentration of double bonds involved in the cross-linking process, based on its established functionality. However, for modified LDH-ATPM, the exact number of modified double bonds could not be determined. This difficulty arises from the ability of clay particle surfaces to bind multiple silane-acrylate agent molecules. To evaluate the network density of nanocomposite hydrogels using LDH-ATPM as the cross-linking agent, swelling behavior serves as a valuable tool. Compared to the initial assessment based on clay-enriched double bonds, swelling behavior can be considered an indirect evaluation method. In this study, simple hydrogels with HEMA/AMPSA molar ratios of 97:3, 95:5, and 100:0 were prepared and evaluated, with MBA molar concentrations ranging from 1% to 3% relative to the monomers. Additionally, nanocomposite hydrogels with the same HEMA/AMPSA molar ratios (97:3, 95:5, and 100:0) but incorporating 1% and 2% LDH-ATPM by weight were also tested. The swelling degree of samples with MBA as the cross-linker was compared to that of nanocomposite hydrogels to evaluate network density. The findings revealed that nanocomposite hydrogels with 3% MBA exhibited a higher swelling degree, corresponding to a reduced cross-linking density compared to simple hydrogels. To enable a more accurate comparison, the MBA molar concentration in simple hydrogels was adjusted to match the swelling degree of nanocomposite hydrogels (see Table 2 for details). All results presented are for samples with monomer molar concentrations of 2 mol/L and 5 mol/L, with HEMA/AMPSA molar ratios of 97:3, 95:5, and 100:0.

**Figure 1:**
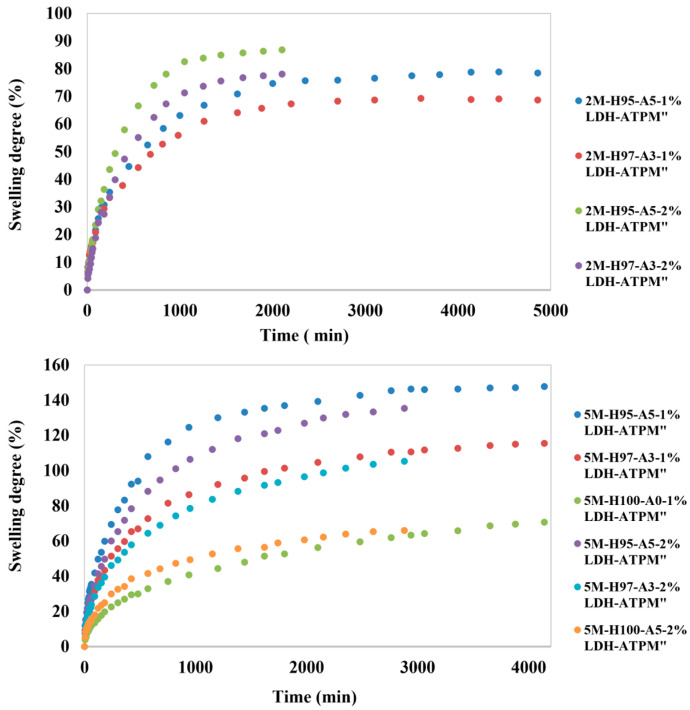
Swelling behavior of nanocomposite hydrogels: top—samples with 2 mol/L monomer concentration; bottom—samples with 5 mol/L monomer concentration.

Furthermore, the swelling analysis provided valuable insights, as outlined below. The nanocomposite hydrogel samples exhibited the expected behavior of an increased swelling degree with higher amounts of the hydrophilic monomer AMPSA (Figure 1). This trend was observed in all cases, with both 1% and 2% LDH-ATPM concentrations and 2 mol/L or 5 mol/L monomer concentrations. The swelling time was significantly shorter for samples containing 2% LDH-ATPM, with durations under 2900 min. In contrast, samples with 1% LDH-ATPM exhibited a substantially longer swelling time, approximately 4100 min. This phenomenon can be attributed to the enhanced chain flexibility resulting from the increased amount of LDH-ATPM, which compensates for the higher cross-linking density. Additionally, it was observed that doubling the concentration of the LDH-ATPM cross-linking agent caused only minimal variation in the swelling degree. The decrease in the swelling degree for nanocomposite hydrogels with 2% modified clay (5 mol/L) was expected to be greater compared to that of classical hydrogel 3D networks [56,57,59,60]. This result may indicate a different network structure in these nanocomposite hydrogels. Furthermore, nanocomposite hydrogels with a 2 mol/L monomer concentration exhibited a less typical behavior, characterized by an increase in swelling degree values with higher amounts of the LDH-ATPM cross-linker. This behavior was partially explained in the case of the 5 mol/L concentration, where 1% and 2% LDH-ATPM exhibited almost identical swelling degree values. For the 2 mol/L samples, an increase in chain flexibility was observed, which was further compensated by the cross-linking density.

For the 5 mol/L samples, the higher molar concentration likely limited the increase in chain flexibility, which may not have fully compensated for the rise in network density. As a result, during swelling, the hydrogel nanocomposites began to crack, restricting their potential biomedical applicability. Consequently, further mechanical, morphological, and biological investigations were conducted exclusively on samples with a 2 mol/L molar concentration.

This behavior is further supported by the findings from swelling kinetic analyses. A comprehensive review of the existing literature highlights various mathematical models and explanations for this phenomenon, including Fickian or first-order transport, less Fickian behavior, anomalous or non-Fickian transport, and second-order transport [61,62,63,64,65]. One of the most widely used mathematical models is the power-law model [66,67,68], in which *n* serves as a critical exponent that explains the diffusion mechanism.

(1) $$\frac{M_{t}}{M_{\alpha }}={k\cdot t}^{n}$$

The power-law equation model, where ***M_t_***—swollen water at time t, ***M_α_***—swollen water at equilibrium, k is a characteristic constant of the hydrogel and n is a characteristic exponent of the molecules transport model. The value of *n* is calculated by plotting the equation in ***log******M_t_/M_α_*** versus in ***log******t*** [36].

Depending on the value of *n*, several models can be considered. For *n* < 0.5, the model is assumed to be Fickian, where Fickian diffusion dominates the transport mechanism. For *n* > 0.5, the model is classified as anomalous or non-Fickian, where chain relaxation governs the transport mechanism [58,64,69,70]. For the nanocomposite samples with a 5 mol/L concentration (Table 3), the following observations were made: (i) Nanocomposite hydrogels containing 1% LDH-ATPM exhibited n exponent values around 0.48, indicating a transition from a purely Fickian domain to a less Fickian regime. In this case, the polymer chain relaxation rate is significantly higher than the water penetration rate. This behavior could be attributed to the high mobility of the polymer chains and the low T_g_ of the system [57]. In contrast, samples with 2% LDH-ATPM exhibited higher *n* exponent values, approaching 0.5, suggesting an apparent increase in local network rigidity. This enhanced rigidity could be attributed to the formation of additional inter- and intramolecular bridges. Moreover, the localized network rigidity may result from the agglomeration of clay layers, a phenomenon particularly evident in the 2% LDH-ATPM samples. The direct influence was attributed to the reduction in the difference between the water penetration rate and the polymer chain relaxation rate. The observed decrease in swelling time for a similar degree of swelling suggests that the increased network rigidity was only apparent.

**Figure 2:**
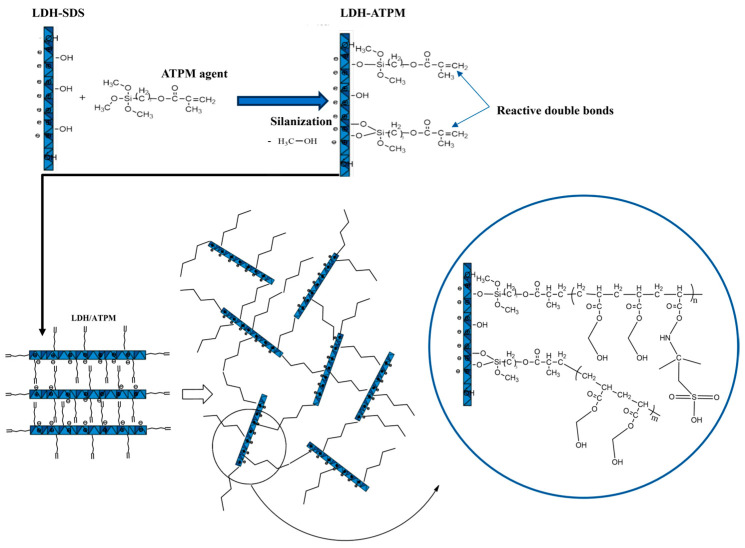
Proposed model of the chemical network structure for nanocomposite hydrogels via the “grafting-through” mechanism involving clay double bond participation.

A model for network formation via a “grafting-through” mechanism was proposed, involving the participation of clay double bonds in the free-radical polymerization of monomers (Figure 2). This phenomenon can be attributed to the rapid accommodation of polymer chains with the solvent, as evidenced by continuous medium rearrangements [64], in contrast to the behavior observed in the 1% LDH-ATPM case. The nanocomposite samples with a 2 M molar concentration (Table 3) exhibited a more pronounced variation in n values between the 1% LDH-ATPM and 2% LDH-ATPM cases. This finding suggests that both apparent and local network rigidity are more pronounced in this case, likely due to the increased number of bridges formed by the cross-linking agent relative to the polymer chain density. In contrast, for samples with a 5 mol/L concentration, no significant increase in the number of bridges was observed. The enhanced apparent rigidity of the network was further supported by the accelerated accommodation to the water solvent, as indicated by a reduced swelling time compared to the 5 mol/L samples. Notably, the swelling degree of 2% LDH-ATPM demonstrated a higher level of rigidity than that of 1% LDH-ATPM. The enhanced flexibility of the novel cross-linked network enables both rapid accommodation to the solvent and continuous structural rearrangements. This network exhibited a distinct response compared to polymers cross-linked by conventional agents. Furthermore, the increased flexibility and chain mobility were highlighted by the network’s inability to establish an elastic force balance to counteract the stress induced by osmotic water diffusion. Additionally, the network exhibited a deficiency in maintaining equilibrium at low values, as indicated by an increase in network density. The results demonstrated that, at least for samples with a 2 mol/L concentration, a higher amount of cross-linking agent (LDH-ATPM) led to a decrease in network bridge density. This outcome is further supported by the observed local agglomeration of clay layers.

The innovative cross-linked network, based on modified clay LDH-ATPM as a cross-linking agent, exhibits a distinctive structural arrangement. A key feature of this system is the clay’s ability to establish multiple cross-linking bridges with adjacent macromolecular chains, a characteristic that sets it apart from conventional polymer 3D networks. This unique interaction enhances network stability, mechanical strength, and structural integrity, making it a promising approach for advanced nanocomposite hydrogels. Morphological characterization and dynamic mechanical analysis indicated that a 1% clay concentration was more suitable than 2% for these nanomaterials when synthesized with a monomer concentration of 2 mol/L. This phenomenon can be attributed to the tendency for layer-specific agglomeration and the reduced dispersion capability at higher clay concentrations, which can negatively impact the uniformity and mechanical performance of the nanocomposite network. This concentration facilitated effective dispersion within the polymer matrix, directly influencing the mechanical behavior of the sample. When the modified clay is regarded as a cross-linking point for inter- and intra-chain bridging, the internal chemical structure of nanocomposite hydrogels with well-dispersed clay layers is depicted in Figure 2.

### 3.3. FTIR Investigation

**Figure 3:**
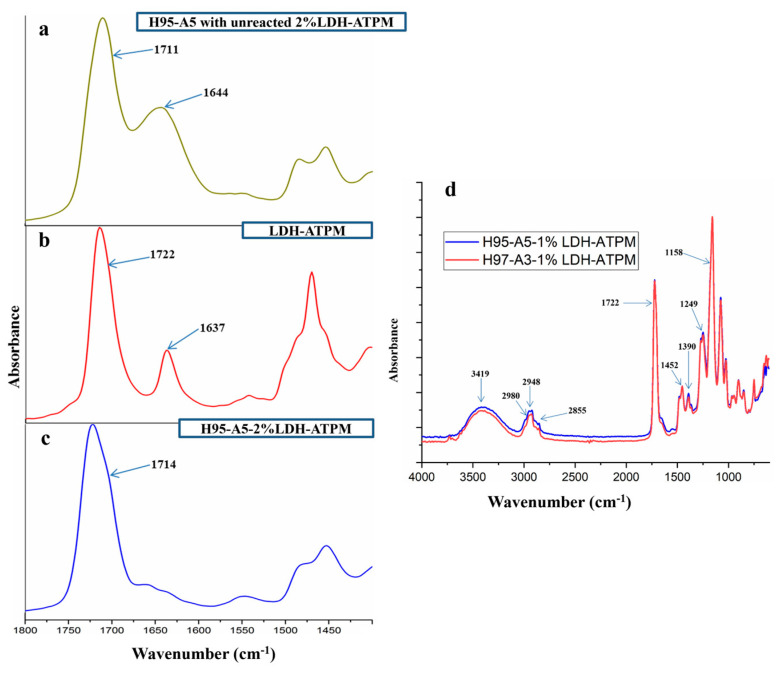
FTIR spectra for: (**a**) nanocomposite with physically dispersed and unreacted LDH-ATPM; (**b**) modified LDH-ATPM; (**c**) nanocomposite with reacted LDH-ATPM; (**d**) comparison of nanocomposites with various monomer ratios (2 mol/L).

The physicochemical characterization of nanocomposite hydrogels was conducted to elucidate the chemical interactions between the double bond-modified LDH-ATPM and the PHEMA/PAMPSA polymer matrix. Figure 3 presents the FTIR spectra for each modified clay, including unreacted and reacted clay, as well as nanocomposite hydrogels with varying HEMA/AMPSA ratios. The FTIR spectra of the nanocomposite hydrogels (Figure 3d) exhibited no significant differences when the HEMA/AMPSA ratio varied between 100/0 and 95/5. Figure 3d revealed the following specific peaks in the FTIR spectra: a peak at 3419 cm^−1^, attributed to O-H stretching from PHEMA units and modified clay; two peaks at 2980 cm^−1^ and 2948 cm^−1^, corresponding to the asymmetric stretching of C-H bonds from methyl and methylene groups, respectively; a more intense peak at 2855 cm^−1^ for the 95/5 ratio, attributed to the symmetric stretching of C-H bonds from methyl groups, which are more prominent in PAMPSA units; a peak at 1722 cm^−1^, assigned to the stretching vibration of C=O bonds from carbonyl groups in PHEMA units and modified clay. A small peak around 1580 cm^−1^ was observed for the 95/5 and 97/3 ratios, which can be attributed to the bending vibration of N-H bonds from PAMPSA units; two peaks at 1452 cm^−1^ and 1390 cm^−1^ corresponded to the bending vibrations of methyl and methylene groups. Additionally, a peak at 1249 cm^−1^ was attributed to the stretching vibrations of C-N and S=O bonds, while a peak at 1158 cm^−1^ was assigned to the stretching vibration of ether C-O-C bonds [71,72,73,74,75]. As shown in Figure 3b, two significant peaks of modified clay LDH-ATPM were identified at 1722 cm⁻¹ and 1637 cm^−1^, corresponding to the stretching vibrations of C=O bonds from carbonyl groups and C=C bonds, respectively. Figure 3c presents the FTIR spectrum of the nanocomposite hydrogel with a 2% LDH-ATPM concentration, aiming to elucidate the interaction between the clay and the polymer matrix. It was hypothesized that this interaction involves the consumption of double bonds during the polymerization process. The disappearance of the peak at 1637 cm^−1^ confirmed the participation and consumption of double bonds from the modified clay in the polymerization reaction of HEMA and AMPSA, leading to the formation of a new cross-linked 3D network. The FTIR spectrum of the nanocomposite sample containing only physically dispersed LDH-ATPM (Figure 3a) exhibited a peak at 1644 cm^−1^, confirming that when the double bonds from the modified clay do not participate in the polymerization reaction, they remain highly visible in the spectrum. This finding further supports the idea that the absence of a specific C=C peak in the nanocomposite spectrum (Figure 3c) is due to its involvement in the polymerization reaction rather than low visibility in the spectrum.

### 3.4. X-Ray Diffraction (XRD) Investigation

**Figure 4:**
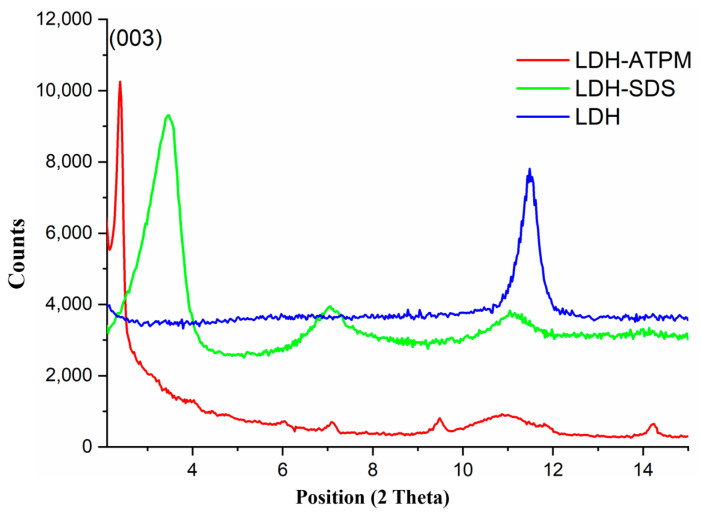
XRD diffractograms of LDH clay species.

The XRD diffractograms (Figure 4) of crude LDH, modified LDH-SDS, and modified LDH-ATPM were analyzed to assess the structural changes induced by the successive chemical modifications of LDH clay. The crude LDH exhibited a primary basal peak at 2θ = 11.47°, corresponding to a specific interlayer d-spacing of approximately 7.7 Å, oriented along the (003) plane. This result suggests the basal reflection of inorganic anions between the clay layers [76]. Modification of the clay with sodium dodecyl sulfate (SDS) caused a shift in the (003) peak toward lower 2θ values at 3.35°, with a corresponding interlayer d-spacing of approximately 26.33 Å. This shift indicates a significant expansion of the interlayer spacing, likely due to the incorporation of SDS molecules between the clay layers. Additionally, two peaks were observed at 2θ = 7.07° and 2θ = 11°, which are characteristic of the crystalline structure of sodium dodecyl sulfate (SDS). Clay modification through the addition of ATPM and anion exchange for SDS removal resulted in a further shift of the basal peak to lower 2θ values at 2.42°, with a corresponding interlayer d-spacing of approximately 36.46 Å. The progressive increase in basal interlayer spacing at each modification step, compared to the previous one, demonstrated a clear structural transformation. This confirmed that the clay was successfully tailored, with carbon double bonds introduced in preparation for the cross-linking reaction [55].

### 3.5. Thermogravimetric Analysis (TGA)

**Figure 5:**
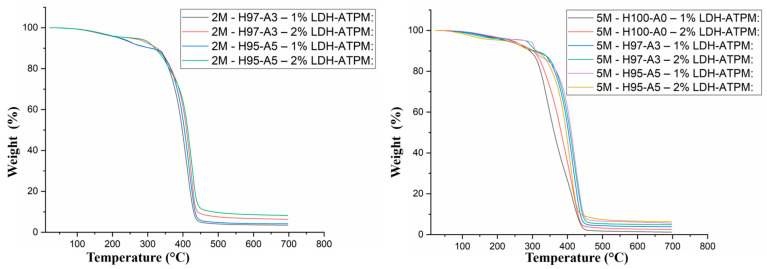
Themorgrams of nanocomposite samples with 2 mol/L and 5 mol/L concentrations.

The TGA analysis (Figure 5) revealed distinct thermal behaviors for samples with 2 mol/L and 5 mol/L concentrations. Samples with a 2 mol/L concentration appeared more homogeneous, exhibiting minimal dependency on the LDH-ATPM concentration around 300°C. The residual mass ranged between 4% and 8%. Notably, samples containing 2% LDH-ATPM exhibited lower weight loss, while no significant differences were observed based on the HEMA-AMPSA ratio. In contrast, samples with 5 mol/L concentration revealed a strong dependency on HEMA-AMPSA ratio and LDH-ATPM concentration. Therefore, samples without AMPSA monomer showed the fastest weight loss while samples with 5% AMPSA revealed the highest thermal stability. This result confirms that samples with a 2 mol/L concentration are more suitable for further applications.

### 3.6. Mechanical Testing

#### 3.6.1. Tensile Test

**Figure 6:**
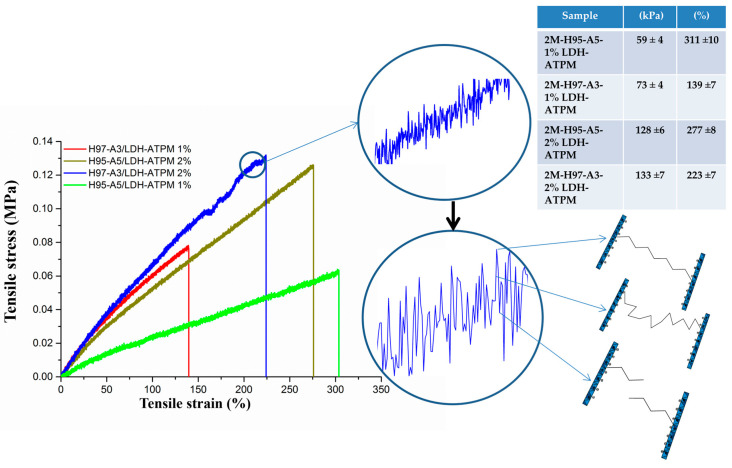
Tensile measurements of nanocomposite hydrogels with various compositions.

It is essential to perform mechanical characterization of nanocomposite hydrogels to evaluate the influence of modified clay and monomer ratio on their mechanical behavior within the polymer matrix. The nanocomposite samples with a 2 mol/L concentration were considered suitable for tensile testing. All four samples were subjected to tensile measurements, prepared as specimens with dimensions of 6 mm in width, 2 mm in thickness, and a 60 mm active area. The results of the tensile stress-strain tests revealed significant variations among samples with different compositions (Figure 6), emphasizing the influence of formulation on mechanical performance. The nanocomposite sample with an H97-A3 ratio and 1% LDH-ATPM exhibited the lowest tensile strain, likely due to the reduced content of the hydrophilic AMPSA monomer, which directly affected the swelling capacity and chain flexibility. This suggests that a lower AMPSA content results in less flexible polymer chains, which are insufficiently adaptable to accommodate the structural rearrangements induced by mechanical stress in the axial direction. Conversely, the H95-A5 sample with 1% LDH-ATPM exhibited a higher tensile strain, which can be attributed to its enhanced chain flexibility due to the increased proportion of hydrophilic AMPSA monomer. However, this was accompanied by lower tensile stress, likely due to the increased water content, which acted as an interposer between the macromolecular chains, weakening physical interactions and thereby reducing overall mechanical strength. The samples with 2% LDH-ATPM exhibited a similar behavior, with comparable values for both tensile stress and tensile strain, suggesting that the increase in clay content did not significantly alter the mechanical response under tensile conditions. This outcome is likely attributable to the quantity of modified clay, which had a more pronounced effect compared to the influence of the monomer ratio. When compared to the 1% LDH-ATPM samples, some notable differences were observed. The 2% LDH-ATPM samples exhibited the expected higher tensile stress, which can be attributed to the doubling of the cross-linking agent, leading to an increase in network rigidity and strength. Furthermore, the tensile test curves obtained in this study displayed a distinctive behavior compared to those reported in the existing literature on analogous hydrogel tests, suggesting that the specific formulation and structural characteristics of these nanocomposite hydrogels influence their mechanical performance in a unique manner [77,78,79]. The curve’s behavior exhibited a local variation in tensile stress with strain, along with an overall linear dependency between tensile stress and strain. This behavior is explained in Figure 5, which illustrates the chain elongation and chain fracture events. The maximum stress point in the local variation corresponds to the peak elongation of the polymer chains involved. The rising segments of the local variation indicate the continuous stretching of the chains, while the lowest points represent chain fracture events, marking the failure of the network structure. Following the fracture of shorter polymer chains, accompanied by the dissipation of elastic energy [80], the sample remained structurally intact. The mechanical load was then transferred to the longer chains, allowing the network to sustain deformation, with this cycle repeating until the material ultimately failed.

#### 3.6.2. Compressive Test

**Figure 7:**
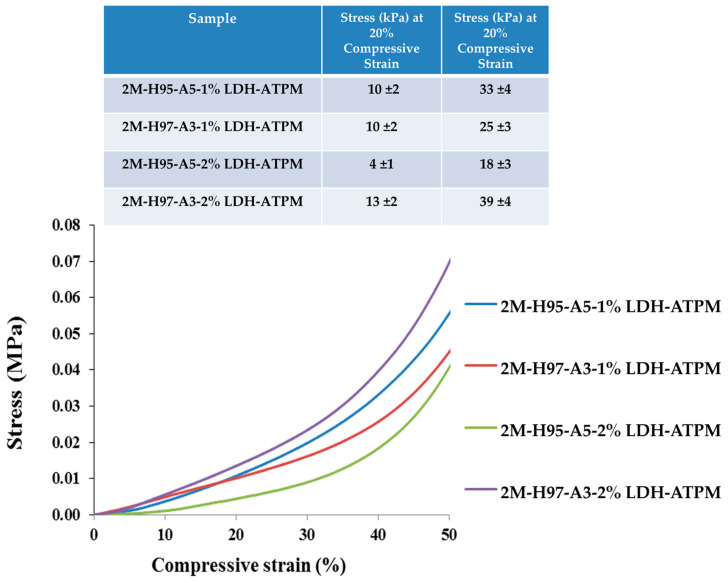
Compression test of nanocomposite hydrogels with various compositions (2 mol/L).

The mechanical characterization of nanocomposite hydrogels was also performed through compression tests. The resulting compression curves (Figure 7) exhibited no fracture events but instead showed a progressive flattening of the samples. Consequently, the relevant compressive strain was analyzed and represented up to 50%, as this range provided meaningful insights into the material’s deformation behavior. Consequently, the quantification of results and differentiation of samples were performed by determining the compressive stress values at 20% and 40% compressive strain, providing a comparative assessment of the materials’ mechanical response under compression. For samples with an H97-A3 ratio, Figure 7 shows an increase in compressive stress at both 20% and 40% compressive strain, which was attributed to the higher amount of cross-linking agent. This increase in cross-linking likely enhanced the network rigidity, resulting in greater resistance to compression. This phenomenon can be attributed to the increased network bridge density, which arises from the higher availability of cross-linker clay. The enhanced cross-linking leads to a more rigid structure, thereby improving the material’s resistance to compression. In contrast, the nanocomposite with an H95-A5 ratio exhibited a decline in compressive stress at both 20% and 40% compressive strain for the sample containing 2% LDH-ATPM. This outcome can be attributed to the reduced dispersal capacity of clay layers compared to 1% LDH-ATPM, which directly influenced the mechanical compressive response of the sample. The diminished dispersion likely resulted in localized agglomeration, reducing the effectiveness of the cross-linking network and weakening the overall structural integrity under compression.

### 3.7. Rheological Measurements

**Figure 8:**
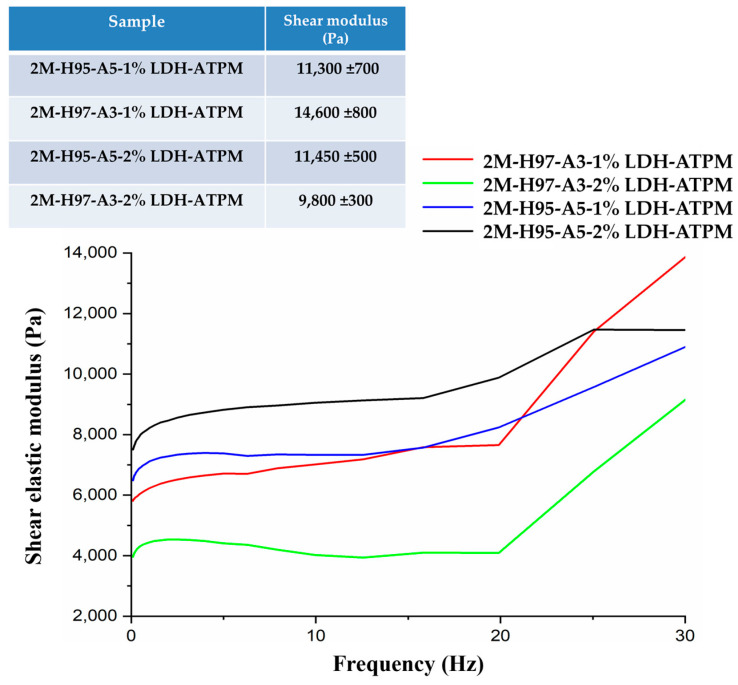
Rheological measurements of nanocomposite hydrogels with various compositions (2 mol/L).

Rheological measurements were conducted on swollen samples at swelling equilibrium to assess their viscoelastic properties. The analysis required stress optimization to ensure the measurements remained within the linear viscoelastic region (LVR), where the material’s response is independent of applied stress. Additionally, the sample’s behavior needed to be dependent only on frequency, ensuring accurate evaluation of its dynamic mechanical properties. The rheological results (Figure 8) revealed a distinct behavior for each nanocomposite composition. The nanocomposite with an H97-A3 ratio exhibited the most significant variation in elastic modulus, suggesting that the absence of the hydrophilic AMPSA monomer limited the composite’s ability to effectively disperse the modified clay layers within the polymer matrix. Furthermore, the incorporation of twice the amount of the cross-linking agent led to inadequate dispersion of the clay layers, directly affecting the mechanical properties of the nanocomposite. The nanocomposite hydrogel with an H95-A5 ratio exhibited similar behavior for both the 1% and 2% LDH-ATPM samples. This phenomenon can be attributed to the increased chain network density, resulting from the higher cross-linker content, being counterbalanced by the enhanced chain flexibility. This increased flexibility was attributed to the contribution of the hydrophilic AMPSA monomer to the polymer matrix, which enhanced polymer segment mobility despite the presence of a denser cross-linked structure.

### 3.8. Morphological Investigation

**Figure 9:**
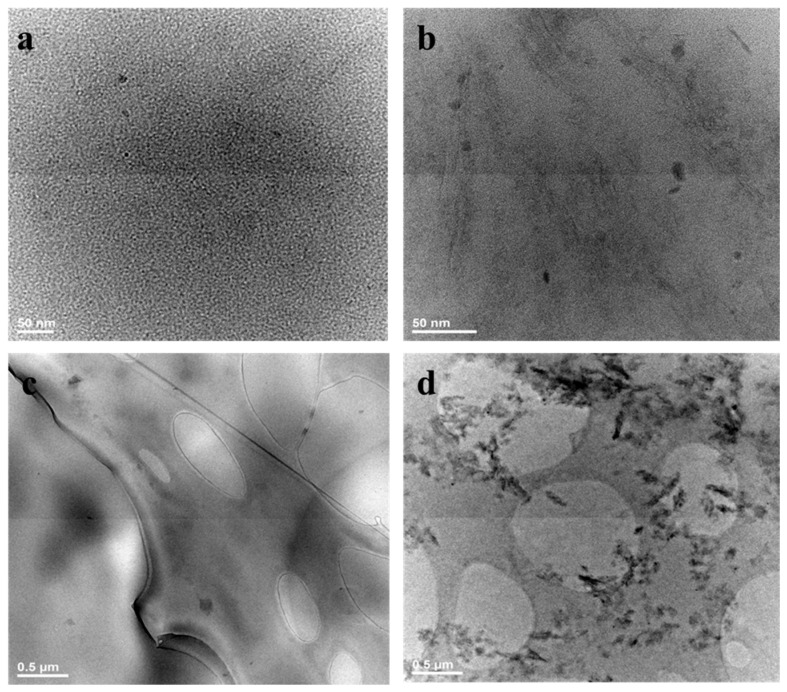
Internal structure for nanocomposite hydrogels (2 mol/L) with dispersion of clay layers: (**a,c**) 1% clay content; (**b**,**d**) 2% clay content.

The morphological characterization of nanocomposite hydrogels was found to be crucial for achieving a comprehensive understanding of both surface topography and internal structuring. This approach enabled the identification of the role of modified LDH-ATPM in the design of 3D cross-linked polymer networks, providing valuable insights into its impact on network formation, dispersion, and overall structural integrity. Morphological characterization using Transmission Electron Microscopy (TEM), Scanning Electron Microscopy (SEM), and micro-CT was performed on samples with a 2 mol/L concentration. This concentration was chosen based on the findings from mechanical and biological analyses, which primarily focused on samples with this molar concentration to assess their structural integrity, dispersion, and network architecture. The internal structure of the nanocomposite hydrogels and the degree of ordering of the clay within the polymeric network were revealed through TEM morphological characterization. Figure 9 presents TEM images highlighting the structural differences between nanocomposite samples with a 95/5 ratio and 1% LDH-ATPM versus 2% LDH-ATPM. The high-resolution images (Figure 9a) of the 1% LDH-ATPM nanocomposite exhibited a high degree of dispersion of the modified clay layers within the polymer matrix. This enhanced dispersion is attributed to the strong bonding between the clay layers and the macromolecular chains, facilitating a more uniform and interconnected network structure. The high dispersion of clay particles, achieved through complete and random distribution within the polymer matrix, was further confirmed in the overview image (Figure 9c), which demonstrated the formation of an exfoliated structure. In contrast, for the nanocomposite sample with 2% LDH (Figure 9b), a well-distributed clay layer was observed within the polymer matrix, however, local clay agglomeration was also present. The overview image (Figure 9d) highlights a mixed structural arrangement, where some regions exhibit well-dispersed clay layers forming an exfoliated structure with a complete and disordered orientation, while other areas show localized clay agglomeration. This configuration can be interpreted as an intercalated structure, characterized by the alternating arrangement of clay layers and polymer chains within the network. The internal morphological structure presented here also provides valuable insights into the swelling behavior and nanocomposite-solvent interactions, helping to explain the relationship between clay dispersion, network integrity, and the material’s response to hydration.

**Figure 10:**
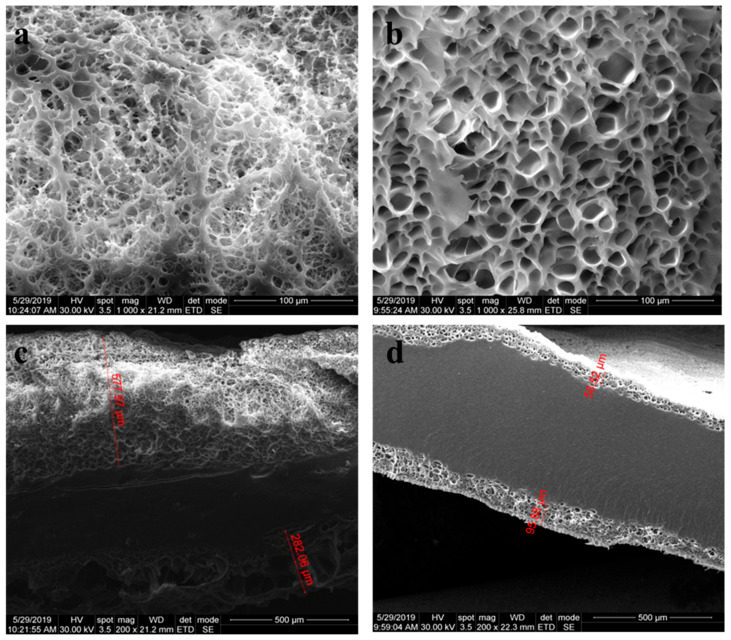
Surface morphology and cross-section of nanocomposite hydrogels (2 mol/L) showing clay layer dispersion: (**a**,**c**) 2% clay content; (**b**,**d**) 1% clay content.

The morphological characterization of nanocomposite hydrogels enabled the subsequent investigation of lyophilized samples with a 2M-H95-A5 ratio, containing 2% LDH-ATPM (Figure 10a) and 1% LDH-ATPM (Figure 10b). To examine both surface topography and internal structure, the samples underwent cryogenic freezing in liquid nitrogen, followed by precision slicing. The 1% LDH-ATPM sample exhibited pores distributed primarily along the sample’s edges, with no perforation through the central region. The porous layer featured surface-open pores oriented toward the exterior, making them particularly suitable for the adhesion of specific cell lines. The pores were found to be well-organized and exhibited a uniform size. The thickness of the surface porous layer remained consistent on both sides of the samples, measuring approximately 50–100 microns. The ratio between the compact center and the porous surface suggests a structural advantage, as the dense central region may enhance the material’s mechanical integrity, while the porous surface facilitates cell adhesion and interaction. The 2% LDH-ATPM sample exhibited pores distributed along the sides, while the central region remained compact. In this case, the thickness of the surface porous layer was greater compared to the compact center, suggesting an increased porosity gradient. The pores exhibited a less organized shape, and a wider size range compared to the 1% LDH-ATPM sample. The formation of pores was observed exclusively toward the surface of the sample, which can be attributed to several factors. The relatively low swelling degree of both samples (approximately 70–90%) suggests a limited distribution of water throughout the sample mass. As a result, the ability of water to form exit pathways by shaping pore structures was restricted. Consequently, the formation of inter- and intramolecular bridges by the modified LDH-ATPM appeared to be sufficiently flexible to accommodate structural rearrangements as water molecules exited the network. The variation of the thickness of the porous layer with the amount of modified clay further corroborates the swelling behaviour by revealing a decrease in network bridge density with LDH-ATPM addition. This behavior can be attributed to the local agglomeration of clay layers. Additionally, despite the decrease in network density and the increase in swelling degree observed in the 2% LDH-ATPM sample, pore formation was not uniform throughout the entire sample mass. This suggests an inconsistent network density, resulting in a gradient of network bridge density from the exterior toward the sample center.

**Figure 11:**
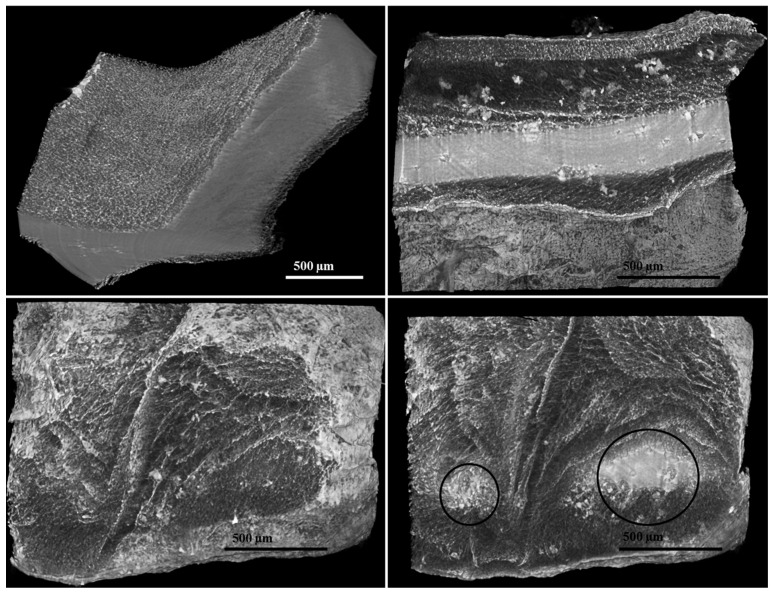
Micro-CT images of nanocomposite hydrogels (2 mol/L) showing clay layer dispersion: top left—1% clay content; top right and bottom—2% clay content.

The internal morphology of lyophilized nanocomposite hydrogels was further examined using X-ray micro-tomography analysis. As illustrated in Figure 11, the images of the 1% LDH-ATPM sample (upper left) and 2% LDH-ATPM sample (upper right) provide insights into the internal structure and pore distribution. The X-ray micro-tomography analysis confirmed the SEM findings, emphasizing the presence of porous surface layers in both 1% and 2% LDH-ATPM samples, with a compact core on each side. Notably, the 1% LDH-ATPM sample exhibited a higher ratio of compact center to porous layer, indicating a preference for a denser internal structure. In contrast, the 2% LDH-ATPM sample showed a greater tendency for porous layer formation, suggesting a more heterogeneous network. The pore distribution in the 2% LDH-ATPM sample is illustrated in Figure 11 (bottom), revealing a sequential layering pattern that ultimately transitions into a compact central region. The obtained images revealed an internal structure characterized by local agglomeration of clay layers, leading to a gradient in network bridge density from the surface toward the center.

Pore size measurements further revealed structural variations influenced by clay concentration. The 1% LDH-ATPM samples exhibited pore sizes of 28 µm (2M-H95-A5-1% LDH-ATPM) and 20 µm (2M-H97-A3-1% LDH-ATPM), indicating a relatively denser network. Conversely, the 2% LDH-ATPM samples showed larger and more variable pore sizes of 38 µm (2M-H95-A5-2% LDH-ATPM) and 16 µm (2M-H97-A5-2% LDH-ATPM), reflecting a more heterogeneous distribution.

### 3.9. Biocompatibility Assessment of the Materials

The selection of human adipose-derived stem cells (hASCs) for cultivation in this study was motivated by their extensively documented capacity to differentiate into various mesenchymal lineages, rendering them particularly suitable for soft tissue engineering applications. Nevertheless, the success of cell adhesion, proliferation, and differentiation is intricately linked to the physicochemical characteristics of the biomaterial, which must be carefully designed to accommodate the specific requirements of hASCs. The functional groups on the material’s surface play a major role in protein adsorption, a fundamental step for effective cell adhesion. The incorporation of sulfonic acid groups from AMPSA increases hydrophilicity, potentially enhancing integrin-mediated adhesion and cellular interactions. Furthermore, the polymer–clay interface in these nanocomposite hydrogels offers supplementary anchoring sites, which may further support cellular attachment. The presence of charged functional groups, such as sulfonate from AMPSA and hydroxyl groups from LDH, plays a crucial role in mediating electrostatic interactions with cells. These interactions impact the adsorption of extracellular matrix (ECM) proteins, thereby regulating integrin binding and influencing cytoskeletal organization.

**Figure 12:**
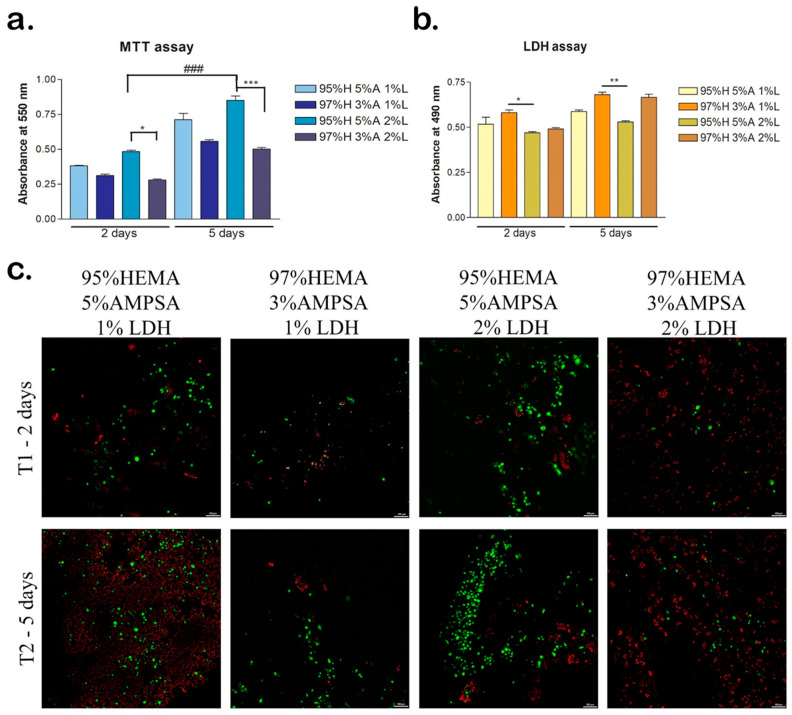
Biocompatibility of the HEMA/AMPSA/LDH materials seeded with hASC after 2 (T1) and 5 days (T2) of cell culture. (**a**) Cell viability and proliferation rate evaluated by MTT test. * *p* < 0.05; ***/### *p* < 0.001; (**b**) Cytotoxicity levels induced by the materials evaluated by LDH test. * *p* < 0.05; ** *p* < 0.01; (**c**) Qualitative LiveDead analysis displaying live cells (green) and nuclei of dead cells (red) of hASCs in HEMA/AMPSA/LDH materials. Scale bar 100 μm.

The MTT cell viability test was used to determine the levels of cell viability in contact with the materials, after 2 and 5 days of cell culture. The materials in contact with the 2 mol/L H95-A5 samples promoted the highest levels of cell viability (Figure 12a). At the 2-day mark, the 2M-H95-A5-2% LDH-ATPM material exhibited the highest levels of cell viability, which were statistically significant (*p* < 0.05) when compared to the H97-A3-2% LDH-ATPM material. This finding suggests that this combination fosters enhanced cell viability and adhesion. This trend persisted at the 5-day mark, with a statistically significant (*p* < 0.001) increase in cell viability observed in the H95-A5-2% LDH-ATPM group compared to the H97-A3-2% LDH-ATPM group. The study revealed that all materials promoted cell proliferation, with a consistent increase in the number of live cells from 2 to 5 days of cell culture. The H95-A5-2% LDH-ATPM induced the highest statistically significant (*p* < 0.001) cell proliferation rate, confirming that this combination of components in the material has a more beneficial effect on cell behaviour than other materials.

The LDH test confirmed the MTT results showing better cytotoxicity levels for the 2M-H95-A5-1% LDH-ATPM and 2M-H95-A5-2% LDH-ATPM materials (Figure 12b). At 2 days, the materials with 2% LDH-ATPM induced similar low cytotoxicity levels suggesting this has a positive influence on cells. The 2M-H97-A3-1% LDH-ATPM material demonstrated the highest level of toxicity at 2 days, exhibiting a statistically significant difference (*p* < 0.05) compared to 2% LDH-ATPM materials, suggesting that this combination of components within the material is more cytotoxic than the other materials. After five days, the 2M-H97-A3 materials exhibited the highest levels of cell death in comparison to the 2M-H95-A5 materials, with statistically significant (*p* < 0.01) higher levels, thereby confirming the results observed at the two-day stage. The Live/Dead assay revealed the presence of live cells (green) and the nuclei of dead cells (red) (Figure 12c). After two days, the 2M-H95-A5-2% LDH-ATPM material exhibited the highest number of live cells, thereby confirming the quantitative results. Additionally, the 2M-H95-A5-1% LDH-ATPM material displayed a substantial number of live cells, suggesting that 2M-H95-A5 materials promote cell viability. In contrast, the 2M-H97-A3 materials exhibited reduced cell viability, as evidenced by a decrease in the number of live cells compared to the 2M-H95-A5 materials. The 2M-H97-A3-2% LDH-ATPM material demonstrated the highest concentration of dead cells, both at 2 and 5 days, suggesting that this material is less conducive to cell survival.

After five days of culture, the same pattern was observed, with an increased number of live cells on 2M-H95-A5 materials compared to all the other compositions. The highest number of live cells and the lowest number of dead cells was observed on 2M-H95-A5-2% LDH-ATPM, suggesting this superior combination of compositions compared to the rest of the materials.

## 4. Conclusions

This study highlighted the development of novel nanocomposite hydrogels based on PHEMA/PAMPSA copolymers, synthesized through a grafting-through process. In this approach, organophilized LDH-ATPM clay served simultaneously as both an inorganic filler and an inorganic cross-linking agent. The LDH-ATPM clay demonstrated its ability to enhance polymer network formation, as evidenced by its distinct mechanical properties and optimized swelling behavior. The study provided key insights into the general limitations of polymer hydrogels’ mechanical flexibility, emphasizing the role of polymer network density in determining mechanical stability and wear resistance through different mechanisms: (i) high network density does not necessarily lead to superior mechanical properties, wear resistance, or high flexibility; rather, excessive cross-linking may reduce elasticity and compromise structural integrity; (ii) network density can be effectively regulated not only by the cross-linking agent concentration but also by the initial monomer concentration, offering an additional parameter for tailoring the mechanical performance of nanocomposite hydrogels. An optimal monomer concentration (HEMA/PAMPSA), the monomer ratio, as well as the nature and concentration of clay, are crucial factors in optimizing the fabrication process for hydrogel nanocomposites with enhanced flexibility and improved wear resistance. The high flexibility of the hydrogel nanocomposites was demonstrated, as three out of four samples (2M-H95-A5-1% LDH-ATPM, 2M-H95-A5-2% LDH-ATPM, and 2M-H97-A3-1% LDH-ATPM) exhibited tensile strain exceeding 200%. Additionally, compressive tests revealed no fracture events, further confirming the good elasticity and structural resilience of these nanocomposite hydrogels. Furthermore, the lyophilization process resulted in an unusual pore distribution and a distinct internal structure within the nanocomposite hydrogels. This approach proved advantageous, as the surface pores facilitated cell adhesion, while the compact hydrogel center provided mechanical stability. Moreover, the developed materials demonstrated biocompatibility, exhibiting high cell viability and low cytotoxicity, making them promising candidates for biomedical applications. The best biocompatibility results were obtained for the 2M-H95-A5-1% LDH-ATPM material, where the optimized component percentages contributed to the overall scaffold quality. Previous studies have highlighted the individual benefits of each material, as well as the advantages of various combinations involving HEMA, PAMPSA, and LDH, further supporting the enhanced performance of this specific formulation. The incorporation of LDH into the material composition significantly enhanced biocompatibility, leading to increased cell viability and reduced cytotoxicity. Furthermore, a higher LDH concentration (2%) improved cellular orientation within the material, promoting cell group formation and fostering a more structured biological response. In conclusion, the developed materials demonstrate strong potential for soft tissue engineering applications, as evidenced by their mechanical resilience, optimized morphology, and favorable biological response.

## Figures and Tables

**Table 1:** Recipes for the preparation of nanocomposite hydrogels.

Sample	Monomer Concentration; 5 mol/L LDH-ATPM Concentration (*w*/*v*) 1%	Monomer Concentration; 5 mol/L LDH-ATPM Concentration (*w*/*v*) 2%	Monomer Concentration; 2 mol/L LDH-ATPM Concentration (*w*/*v*) 1%	Monomer Concentration; 2 mol/L LDH-ATPM Concentration (*w*/*v*) 2%
Monomer ratio (mol/mol): 100–0	5M-H100-A0-1% LDH-ATPM	5M-H100-A0-2% LDH-ATPM	No crosslinking	No crosslinking
Monomer ratio (mol/mol): 97–3	5M-H97-A3-1% LDH-ATPM	5M-H97-A3-2% LDH-ATPM	2M-H97-A3-1% LDH-ATPM	2M-H97-A3-2% LDH-ATPM
Monomer ratio (mol/mol): 95–5	5M-H95-A5-1% LDH-ATPM	5M-H95-A5-2% LDH-ATPM	2M-H95-A5-1% LDH-ATPM	2M-H95-A5-2% LDH-ATPM

Example: 5M-H100-A0-1% LDH-ATPM: 5 mol/L monomer concentration; 100% HEMA monomer; 0% AMPSA monomer; 1% (*w*/*w*) clay LDH-ATPM concentration.

**Table 2:** Evaluation of network density through comparison of swelling degrees in samples cross-linked with LDH-ATPM and the classical MBA agent.

Sample	LDH-ATPM	MBA Equivalent	Sample	LDH-ATPM	MBA Equivalent
2M-H95-A5	1%	2.5%	2M-H95-A5	2%	1.6%
2M-H97-A3	1%	2.75%	2M-H97-A3	2%	2.5%
2M-H100-A0	-	-	2M-H100-A0	-	-
5M-H95-A5	1%	0.8%	5M-H95-A5	2%	0.9%
5M-H97-A3	1%	1.1%	5M-H97-A3	2%	1.1%
5M-H100-A0	1%	2.6%	5M-H100-A0	2%	2.75%

**Table 3:** Swelling degree, swelling time, and transport exponent for nanocomposite hydrogels with 5 mol/L and 2 mol/L monomer concentrations.

Sample	Time (minutes)	Swelling Degree (%)	n	Sample	Time (minute)	Swelling Degree (%)	n
2M-H95-A5-1% LDH-ATPM	4920	78 ± 4	0.4130 ± 0.002	2M-H95-A5-2% LDH-ATPM	2140	86 ± 5	0.6103 ± 0.002
2M-H97-A3-1% LDH-ATPM	4920	67 ± 5	0.4130 ± 0.002	2M-H97-A3-2% LDH-ATPM	2020	78 ± 5	0.601 ± 0.002
2M-H100-A0-1% LDH-ATPM	-	-	-	2M-H100-A0-2% LDH-ATPM	-	-	-
5M-H95-A5-1% LDH-ATPM	4140	145 ± 10	0.4826 ± 0.003	5M-H95-A5-2% LDH-ATPM	2880	137 ± 8	0.4978 ± 0.004
5M-H97-A3-1% LDH-ATPM	4140	112 ± 8	0.4815 ± 0.003	5M-H97-A3-2% LDH-ATPM	2880	107 ± 6	0.4961 ± 0.003
5M-H100-A0-1% LDH-ATPM	4140	74 ± 4	0.4808 ± 0.005	5M-H100-A0-2% LDH-ATPM	2880	68 ± 5	0.4961 ± 0.004

## Data Availability

The original contributions presented in this study are included in the article. Further inquiries can be directed to the corresponding author.
